# Treatment outcomes and late toxicities in patients with embryonal central nervous system tumors

**DOI:** 10.1186/1748-717X-9-201

**Published:** 2014-09-11

**Authors:** Kazumasa Odagiri, Motoko Omura, Masaharu Hata, Noriko Aida, Tetsu Niwa, Hiroaki Goto, Susumu Ito, Masanori Adachi, Haruyasu Yoshida, Hiroko Yuki, Tomio Inoue

**Affiliations:** Department of Radiation Oncology, Yokohama Municipal Citizen’s Hospital, 56 Okazawa-cho, Hodogaya-ku, Yokohama, Kanagawa Japan; Department of Radiology, Kanagawa Children’s Medical Center, 2-138-4 Mutsukawa, Minami-ku, Yokohama, Kanagawa Japan; Department of Radiation Oncology, Shonan Kamakura General Hospital, 1370-1 Okamoto, Kamakura, Kanagawa Japan; Department of Radiology, Yokohama City University Graduate School of Medicine, 3-9 Fukuura, Kanazawa-ku, Yokohama, Kanagawa Japan; Department of Radiology, Tokai University School of Medicine, 143 Shimokasuya, Isehara, Kanagawa Japan; Division of Hemato-Oncology/Regeneration Medicine, Kanagawa Children’s Medical Center, 2-138-4 Mutsukawa, Minami-ku, Yokohama, Kanagawa Japan; Department of Neurosurgery, Kanagawa Children’s Medical Center, 2-138-4 Mutsukawa, Minami-ku, Yokohama, Kanagawa Japan; Department of Endocrinology and Metabolism, Kanagawa Children’s Medical Center, 2-138-4 Mutsukawa, Minami-ku, Yokohama, Kanagawa Japan; Department of Radiation Technology, Kanagawa Children’s Medical Center, 2-138-4 Mutsukawa, Minami-ku, Yokohama, Kanagawa Japan

**Keywords:** Embryonal central nervous system tumors, Medulloblastoma, Supratentorial primitive neuroectodermal tumor, Atypical teratoid/rhabdoid tumor, Pineoblastoma, Growth height, Hypothyroidism, Ototoxicity, Cognitive function

## Abstract

**Background:**

Standard treatment strategies for embryonal central nervous system (CNS) tumors have not yet been established. We treated these tumors using an original chemoradiation therapy protocol; the clinical outcomes and toxicities were retrospectively evaluated.

**Methods:**

Twenty-four patients were enrolled including sixteen with medulloblastoma, four with supratentorial primitive neuroectodermal tumor (sPNET), three with atypical teratoid/rhabdoid tumor, and one with pineoblastoma. Immediately after diagnosis, all patients underwent surgery initially. They were then categorized as high- or average-risk groups independent of tumor type/pathogenesis. The average-risk group included patients who were aged ≥3 years at diagnosis, had non-metastatic disease at diagnosis (M0), and had undergone gross total resection. Other patients were categorized as the high-risk group; this group received more intensive treatment than the average-risk group, including high-dose chemotherapy with autologous stem-cell transplantation. All patients received craniospinal irradiation (CSI). The CSI dose was 23.4 Gy for M0 patients aged ≥5 years, 18 Gy for M0 patients aged <5 years, and 30–36 Gy for all patients with M + disease. The total dose to the primary tumor bed was 54 Gy.

**Results:**

The median follow-up time was 73.5 (range, 19–118) months. The 5-year progression-free survival (PFS) and overall survival (OS) rates were 71.1 and 88.9%, respectively in the average-risk group (n = 9) and 66.7 and 71.1%, respectively in the high-risk group (n = 15). The PFS and OS rates were not significantly different between the average- and high-risk groups. In patients with medulloblastoma only, these rates were also not significantly different between the average- and high-risk groups. Three of four patients with sPNET were disease free. The height standard deviation score (SDS) was significantly decreased at the last assessment relative to that at diagnosis (*P* < 0.0001). The latest median height SDS was -1.6 (range, 0.9 to -4.8), and the latest median full-scale intelligence quotient (FSIQ) score was 86 (range, 59–128). The CSI doses and age at the start of radiation therapy did not influence clinical outcomes, height SDSs, and FSIQ scores.

**Conclusions:**

Our original protocol for patients with embryonal CNS tumors was feasible and yielded favorable clinical outcomes.

## Background

Embryonal central nervous system (CNS) tumors include medulloblastoma (MB), supratentorial primitive neuroectodermal tumor (sPNET), atypical teratoid/rhabdoid tumor (AT/RT), pineoblastoma and others. As recurrence with leptomeningeal dissemination is commonly observed, prophylactic craniospinal irradiation (CSI) is essential for initial treatment of these diseases. Radiation therapy (RT) is generally avoided in patients aged <3 years to prevent the risk of long-term neurocognitive sequelae [1–4].

MB is the most frequent primary solid CNS tumor in children, accounting for 10–20% of CNS neoplasms and for approximately 40% of all tumors arising from the posterior fossa. It can occur in any age group, but the peak incidence is between the ages of 3 and 7 years. Medulloblastoma patients are generally categorized as being in average- and high-risk groups as defined by the Children’s Cancer Study Group (CCSG). The average-risk group is aged ≥3 years at diagnosis, have non-metastatic disease at diagnosis (M0), and have undergone total or nearly total resection. Other patients are categorized as being in the high-risk group [5]. With average-risk MB, the standard dose of CSI was approximately 36 Gy until around the year 2000 [6]. Several clinical trials have been undertaken to reduce the late adverse effects caused by CSI. As a result, reduced-dose CSI involving a total dose of 23.4 Gy has become the standard treatment for the average-risk group [7]. Although the optimal treatment strategy in the high-risk group has not yet been defined, around 36 Gy is considered as the standard dose for CSI [8]. There is now sufficient evidence that boost radiation confined to the tumor bed after CSI, at doses up to 54–55.8 Gy, is adequate for both risk groups [7, 9].

sPNET and pineoblastoma are the two most common subtypes of supratentorial embryonal tumors. The clinical outcomes for sPNET and pineoblastoma have generally been inferior to those for MB. In approximately one third of patients, leptomeningeal dissemination is apparent at the time of diagnosis or initial recurrence. Usually, CSI at 36 Gy with a boost of 54–55.8 Gy to the tumor bed are administered in the same manner as in high-risk MB patients [10, 11].

AT/RT accounts for 1–2% of all pediatric brain tumors; however, 10–20% occur in patients aged <3 years at diagnosis. Up to 15–25% of patients show leptomeningeal dissemination at diagnosis. The overall survival (OS) is the worst among all embryonal CNS tumors. In particular, patients aged <3 years have shorter survival times than others [12].

Recently, high-risk MB, sPNET, AT/RT, and pineoblastoma have been treated using high-dose chemotherapy (HDC) with autologous stem-cell transplantation (ASCT) just after induction chemotherapy [9, 12, 13]. Such aggressive chemotherapy could compensate for the dose reduction in CSI [14].

In the present study, we first categorized all patients with embryonal CNS tumors as high-risk or average-risk groups independent of tumor type/pathogenesis referring to the risk classification which applied for MB, as defined by the CCSG [5].

Immediately after surgery, the average-risk group received conventional chemotherapy, while the high-risk group received a more intensive chemotherapy regimen including HDC with ASCT. All M0 patients aged ≥5 and <5 years underwent reduced-dose CSI at doses of 23.4 and 18 Gy, respectively independent of risk group and tumor type. Therefore, the originality of our protocol is that reduced-dose CSI was administered to all patients with M0 disease, not only to the average-risk MB patients but also to the high-risk MB, sPNET, AT/RT, and pineoblastoma patients. The purpose of this study was to retrospectively evaluate the clinical outcomes and toxicities.

## Methods

### Patients and risk classification

Between March 2003 and January 2011, 26 consecutive patients with newly diagnosed embryonal CNS tumors were treated in our institution. Two patients were not able to undergo the initial standard treatment including RT. In one patient, CSI was cancelled after the delivery of 6 Gy because of respiratory insufficiency caused by pulmonary hypertension. The other patient could not undergo CSI because of intractable epilepsy. The remaining 24 patients were investigated in this study; their medical records were retrospectively reviewed. This retrospective study was performed according to the guidelines of the Kanagawa Children’s Medical Center Ethical Committee. All patients underwent magnetic resonance imaging (MRI) of the brain and whole spine and cerebrospinal fluid examination. Immediately after diagnosis, all patients underwent surgery initially. The extent of surgical resection of the primary tumor was defined as gross tumor resection (GTR), or non-GTR based on review of the postoperative MRI and the surgeon’s intraoperative assessment.

We categorized all patients with embryonal CNS tumors as high-risk or average-risk groups referring to the risk classification which applied for MB as defined by the CCSG [5]. The average-risk group included patients with all of the following characteristics: were aged ≥3 years at diagnosis; had M0 disease; and underwent GTR. Other patients were categorized as being in the high-risk group.

### Treatment after surgery

**Figure 1:**
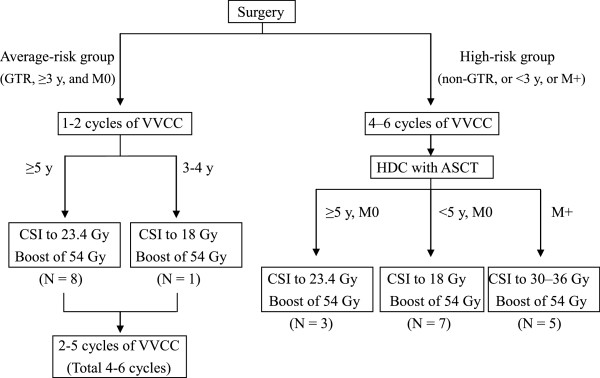
**Schema of the chemoradiation therapy protocol.** ASCT = autologous stem-cell transplantation; CSI = craniospinal irradiation; GTR = gross total resection; HDC = high-dose chemotherapy; VVCC = vincristine (1.5 mg/m^2^, day 1), etoposide (100 mg/m^2^, days 1–5), cisplatin (20 mg/m^2^, days 1–5), and cyclophosphamide (1.2 g/m^2^, days 1–2); y = years.

The treatment protocol is shown in Figure 1. All patients in both the average- and high-risk groups received 4–6 cycles of chemotherapy including vincristine (1.5 mg/m^2^, day 1), etoposide (100 mg/m^2^, days 1–5), cisplatin (20 mg/m^2^, days 1–5), and cyclophosphamide (1.2 g/m^2^, days 1 and 2) (VVCC). For the average-risk group, RT was administered after 1 or 2 cycles of VVCC and then the remaining 2–5 cycles were given. In contrast, for the high-risk group, RT was undertaken after all courses of chemotherapy had been completed. In addition, for patients in the high-risk group, HDC with ASCT was administered after VVCC. HDC consisted of a total dose of 800 mg/m^2^ of TEPA and a total dose of 280 mg/m^2^ of melphalan on days -12, -11, -5 and -4. All patients aged <3 years received maintenance chemotherapy including cyclophosphamide (250 mg/m^2^, days 1–5) and topotecan (0.75 mg/m^2^, days 1–5) after the chemotherapy mentioned above until they were 3 years old. The chemotherapy protocols were modified in a few patients. When disease recurrence or progression occurred, salvage chemotherapy was performed.

Treatment planning for RT was performed using an X-ray simulator until a CT-based planning system was introduced in 2006. All patients were irradiated using a linear accelerator based photon beam. A 6-megavolt (MV) beam was used for CSI and 6- or 10-MV beams were used for the radiation boost at the primary site. The daily fraction size for CSI was mainly 1.8 Gy. Four patients received CSI in 1.5 Gy/fraction and one patient received CSI in 1.7 Gy/fraction. In all patients, the daily fraction size for the boost was 1.8 Gy. Five fractions were given per week. CSI was administered to all patients. It was performed in the supine position using parallel-opposed lateral cranial fields that abutted a posterior spinal field. Two types of junctions were used, termed the ‘low cranial-spinal junction’ and the ‘high cranial-spinal junction’. These two junctions were changed daily. The former was set just above the shoulders and the latter was set 2 cm above the former junction. The lower border of the spinal field was set at the S3–S4 junction.

As shown in Figure 1, the total CSI dose for M0 patients aged ≥5 years at the start of RT was 23.4 Gy, and for those aged <5 years it was 18 Gy. The total dose for patients with metastasis (M+; M1–3) was 30–36 Gy. A boost dose of 54–55.8 Gy was delivered to the primary tumor bed with a limited margin of 1–2 cm. It is well known that RT involving the CNS can cause severe neurological morbidity (i.e. a reduction in the intelligence quotient (IQ), cognitive deficits, and neuroendocrine dysfunctions) in children younger than 3 years [1–4]. Therefore, if patients were aged <3 years at diagnosis, RT was usually postponed until they had turned 3 years, as long as disease was controlled by the chemotherapy mentioned above.

### Statistical analysis

Progression-free survival (PFS) and OS rates were calculated according to the Kaplan–Meier method. PFS reflected the interval between diagnosis and clinical and/or radiological progression. OS was the interval between diagnosis and death. Comparisons between survival curves were made using the log-rank test. Statistical significance was accepted for *P*-values <0.05.

## Results

### Patient demographics

Patient demographics and outcomes are shown in Tables 1 and 2. Of the 24 eligible patients, 16 were male and eight were female. Median age at diagnosis was 5.7 (range, 0.3–16.0) years. There were 16 MBs, four sPNETs, three AT/RTs, and one pineoblastoma. Nine and 15 patients were classified as being in average- and high-risk groups. Coincidentally, the average-risk group consisted of only patients with MB patients, while the high-risk group consisted of patients with a variety of diseases. In 10 patients aged <3 years at diagnosis, four received RT before 3 years because their treatment had failed to control the disease.

**Table 1:** **Clinical and treatment-related information for patients with average-risk embryonal central nervous system tumors**

	Patient no.	Sex	Age at diagnosis (years)	Age at the start of RT (years)	CSI dose (Gy)	Boost dose (Gy)	Follow-up time from diagnosis (months)	Disease progression after RT	Age at the last assessment (years)	Status	Initial height (SDS)	Latest height (SDS)	Hypothyroidism	Ototoxicity (Brock grade)	Initial FSIQ	Latest FSIQ
Medulloblastoma																
	1	F	3.5	3.3	18	54	39	–	6.8	NED	-1.1	-2.2	Subclinical	NE	NE	71
	2	M	4.9	5.0	23	54	33	LR	7.7	DOD	-1.5	-3.5	NE	4	NE	NE
	3	M	6.7	6.9	26	54.3	76	–	13.0	NED	+1.3	+0.4	–	1	98	59
	4	M	7.1	7.3	23	54	94	–	14.9	NED	-1.8	-2.5	Subclinical	0	112	110
	5	M	7.2	7.3	23	54	98	LF	15.3	AWD	-0.5	-2.6	Subclinical	2	135	128
	6	M	9.4	9.6	23	54	34	–	12.3	NED	+1.0	+0.1	NE	2	NE	NE
	7	M	10.2	10.4	23	54	48	–	14.2	NED	+1.2	-0.2	–	1	81	82
	8	F	12.8	12.9	23	55.8	94	–	20.6	NED	+1.0	+0.2	NE	2	NE	NE
	9	F	16.0	16.3	23	54	79	–	22.6	NED	+1.0	+0.9	Subclinical	NE	NE	NE

*Abbreviations*: *AWD* alive with disease, *CSI* craniospinal irradiation, *DOD* death of disease, *FSIQ* full-scale intelligence quotient; *LF* local failure; *LR* leptomeningeal recurrence; *NE* not evaluated, *NED* no evidence of disease, *RT* radiation therapy, *SDS* standard deviation score.

**Table 2:** **Clinical and treatment-related information for patients with high-risk embryonal central nervous system tumors**

	Patient no.	Sex	Age at diagnosis (years)	Primary tumor site	M stage	Extent of initial surgery in primary site	Age at the start of RT (years)	CSI dose (Gy)	Boost dose (Gy)	Administration of HDC + ASCT before RT	Maintenance therapy before RT	Status before RT	Follow-up time from diagnosis (months)	Disease progression after RT	Age at the last assessment (years)	Status	Initial height (SDS)	Latest height (SDS)	Hypothyroidism	Ototoxicity (Brock grade)	Initial FSIQ	Latest FSIQ
Medulloblastoma																						
	10	M	1.9	I	M0	GTR	2.3	18	54	Yes	–	PD^†^	28	LR	4.3	DOD	NE	NE	NE	0	NE	NE
	11	M	2.7	I	M0	non-GTR	3.3	18	50.4	Yes	–	CR	109	–	11.8	NED	-0.3	-1.8	–	0	NE	95
	12	M	6.3	I	M1	GTR	6.4	31	54	No	–	CR	92	–	13.9	NED	0	-1.5	Subclinical	0	88	86
	13	F	8.1	I	M3	GTR	8.8	36	54	Yes	–	CR	23	–	10.0	NED	0	-0.7	NE	0	NE	96
	14	F	10.2	I	M0	non-GTR	10.9	23	55.8	Yes	–	PR^‡^	109	–	19.3	SD	+0.5	-1.6	Subclinical	4	101	82
	15	M	10.3	I	M0	non-GTR	11.0	23	54	Yes	–	CR	50	–	14.4	NED	-2.4	-4.1	Secondary	0	NE	79
	16	M	11.8	I	M1	GTR	13.5	23	54	Yes	–	CR	83	–	19.8	NED	+0.4	-0.7	–	0	102	NE
sPNET																						
	17	M	2.3	S	M0	GTR	3.0	18	54	Yes	CT, ICE	PD^†^	28	LF	4.6	DOD	NE	NE	NE	NE	94	NE
	18	M	2.4	S	M0	GTR	3.3	18	54	Yes	CT	CR	92	–	10.1	NED	-0.5	-1.9	Subclinical	0	128	107
	19	F	2.4	S	M0	GTR	3.4	18	54	Yes	CT	CR	29	–	4.8	NED	+1.1	-0.1	Subclinical	NE	86	NE
	20	F	2.5	S	M0	Non-GTR	3.8	18	54	No	CT	CR	66	–	8.0	NED	-0.9	-2.4	–	NE	72	77
AT/RT																						
	21	M	0.3	I	M3	GTR	1.5	30	51	Yes	–	CR	71	LR	6.3	DOD	-1.0	-4.8	NE	2	NE	NE
	22	F	1.3	S	M0	Non-GTR	2.6	18	54	Yes	IFNß	CR	118		11.2	NED	-0.4	-1.1	Subclinical	2	75	62
	23	M	2.8	I	M3	Non-GTR	3.3	31	39.6*	Yes	–	PR^§^	46	LR	6.7	DOD	NE	NE	NE	0	102	88
Pineoblastoma																						
	24	M	1.3	S	M3	Non-GTR	2.3	30	49.8	Yes	CT, MTX	PD^¶^	19	LR	2.8	DOD	NE	NE	NE	NE	NE	NE

*Abbreviations: ASCT* autologous stem-cell transplantation, *AT/RT* atypical teratoid/rhabdoid tumor, *CR* complete response, *CSI* craniospinal irradiation, *CT* cyclophosphamide and topotecan, *DOD* death of disease, *FSIQ* full-scale intelligence, *GTR* gross total resection, *HDC* high-dose chemotherapy, *I* infratentorial, *ICE* ifosfamide, carboplatin and etoposide, *IFN* interferon, *LF* local failure, *LR* leptomeningeal recurrence, *MTX* methotrexate, *NE* not evaluated, *NED* no evidence of disease, *PD* progression disease, *PR* Partial response, *RT* radiation therapy, *S* supratentorial, *SD* stable disease, *SDS* standard deviation score, *sPNET* supratentorial primitive ectodermal tumor

*Boost RT was limited to spinal lesion of Th9-S2.

^†^Local failure.

^‡^Residual primary tumor.

^§^Residual spinal seeding.

^¶^Leptomeningeal recurrence.

### Treatment outcomes

**Figure 2:**
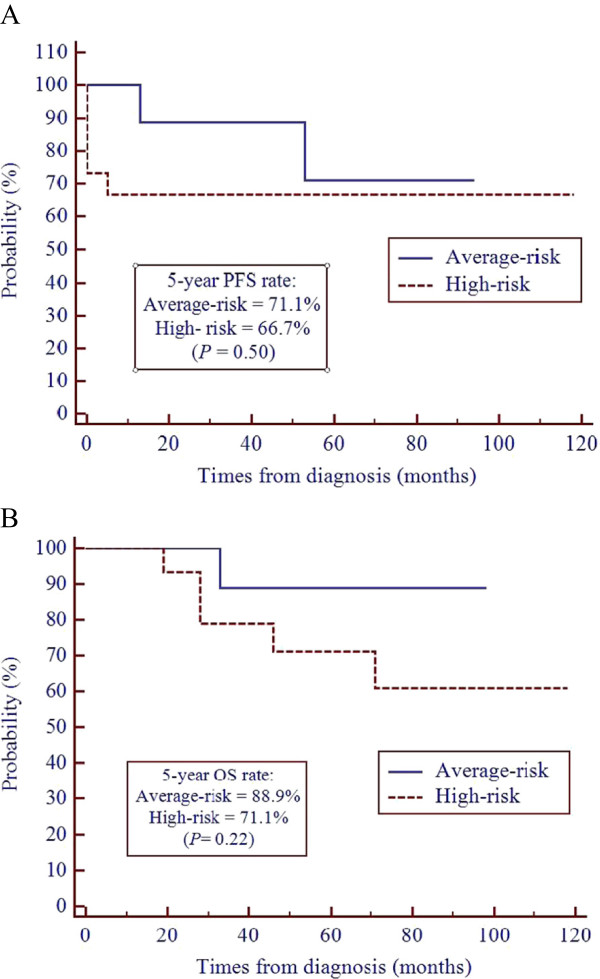
**Progression-free survival (A) and overall survival (B) of patients with average- and high-risk embryonal central nervous system tumors.**

**Figure 3:**
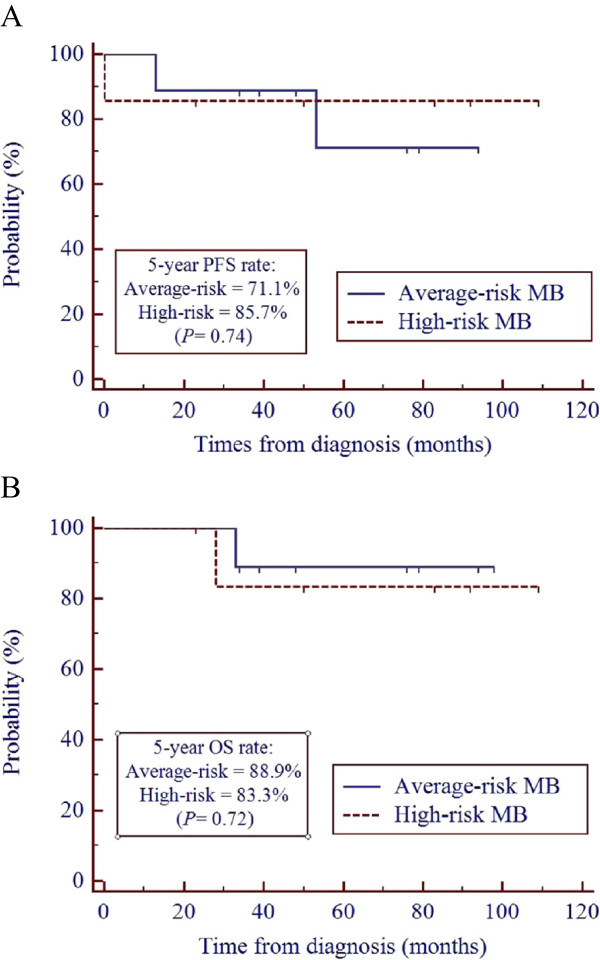
**Progression-free survival (A) and overall survival (B) of patients with medulloblastoma.** MB = medulloblastoma.

The median follow-up time was 73.5 (range, 19–118) months from diagnosis and 57.5 (range, 5–103) months from the start of RT. In the average-risk group (n = 9), the 5-year PFS and OS rates were 71.1 and 88.9%, respectively. In the high-risk group (n = 15), the 5-year PFS and OS rates were 66.7 and 71.1%, respectively. The PFS and OS rates did not differ significantly between the average- and high-risk groups (Figure 2). If limited to MB patients, in the average-risk group (n = 9) the 5-year PFS and OS rates were 71.1 and 88.9%, respectively, and in the high-risk group (n = 7) the 5-year PFS and OS rates were 85.7 and 83.3%, respectively. For MB patients, the PFS and OS rates were not significantly different between the average- and high-risk groups (Figure 3).

As shown in Table 2, none of the patients with sPNET had M + disease. One patient died of local failure, whereas the remaining three patients were disease free until the last assessment. In three patients with AT/RT, two had M3 disease and died of leptomeningeal dissemination. The remaining M0 patient remained disease free. One patient with pineoblastoma had M3 disease and died of leptomeningeal dissemination.

Between patients who received CSI at doses of 18 Gy (n = 8) and 23.4 Gy (n = 10), no significant difference was found in the 5-year PFS rate (75.0 vs. 75.0%; *P* = 0.73) or the OS rate (75.0 vs. 90.0%; *P* = 0.35). There was no treatment-related death.

### Growth height

**Figure 4:**
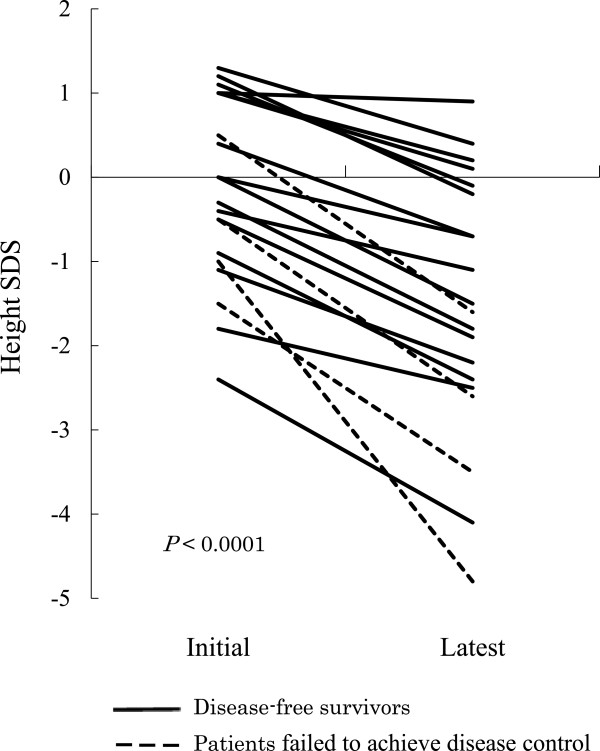
**Differences in height standard deviation score (SDS) at diagnosis and at the last assessment.**

In 20 of the 24 patients, continuous growth charts were available at least ≥2 years after initial diagnosis. The height of these 20 patients was evaluated using a standard deviation score (SDS) from the age-matched mean value in the normal Japanese population [15]. The median age at the latest assessment was 11.5 (range, 4–22) years. The median time between initial assessment at diagnosis and the latest assessment was 71.5 (range, 24–109) months. The data for each patient is detailed in Tables 1 and 2. Figure 4 shows the differences in the initial and latest height SDS. The median latest height SDS was -1.6 (range, 0.9 to -4.8). There were seven patients with a SDS < - 2. The latest height SDS was significantly decreased relative to the initial height SDS (*P* < 0.0001). The median loss of height SDS was 1.3 (range, 0.1–3.8).The 20 patients included four who failed to achieve disease control (Patients 2, 5, 14, and 21, as shown by the dotted lines in Figure 4). Three of these four patients had a latest height SDS < - 2. When these four patients were eliminated, the median latest height SDS was -0.9.

The doses of CSI (>18 Gy vs. ≤18 Gy) and age at the start of RT (≥5 years vs. <5 years) did not influence the latest height SDS or the loss of height SDS.

Three patients received growth hormone replacement therapy.

### Hypothyroidism

Direct irradiation of the thyroid could cause primary hypothyroidism. This is defined as low free thyroxine (T4) and elevated thyroid-stimulating hormone (TSH) levels. The subclinical hypothyroidism was defined as a normal free T4 level but with a high TSH level. In addition, the damage to the hypothalamic-pituitary axis could also cause secondary hypothyroidism defined as low free T4 and TSH levels.

The levels of T4 and TSH were assessed in 15 patients and the data for each patient is presented in Tables 1 and 2. Nine patients (60%) developed subclinical hypothyroidism and three of these received thyroid hormone replacement therapy (Patients 14, 18, and 22). Patient 15 developed secondary hypothyroidism immediately after surgery. Neither age at the start of RT (≥5 years vs. <5 years) nor CSI dose (>18 Gy vs. ≤18 Gy) affected thyroid function.

We retrospectively assessed the radiation doses to the thyroid gland in 10 patients whose CT planning data were available. Mean doses to the thyroid gland were 11.6–24.7 Gy (median, 18.5 Gy). Subclinical hypothyroidism was observed in two patients (Patients 1 and 19) who received CSI at a dose of only 18 Gy and their estimated mean doses to the thyroid gland were 11.6 and 11.8 Gy. Posterior spinal fields for CSI using a high cranial-spinal junction usually included the whole volume of the thyroid gland; whereas, using a low cranial-spinal junction the thyroid volumes varied among individual cases. However, even when using a low cranial-spinal junction, there were no cases where the posterior spinal field could completely exclude the thyroid gland.

### Ototoxicity

We used the Brock criteria, which are designed to evaluate cisplatin-related ototoxicity, to analyze hearing status [16]. Brock grade 0 is <40 dB at all frequencies. Grades 1, 2, 3, and 4 are ≥40 dB at 8000, >4000, 2000, and 1000 Hz, respectively. Audiograms were available for 14 of the 24 patients and the data for each patient is detailed in Tables 1 and 2. Two patients had Brock grade 1 ototoxicity, five had grade 2, and two had grade 4. Seven patients (50%) suffered hearing loss of Brock grade ≥2, which often required audiological intervention [17, 18]. Of note, hearing loss of Brock grade 2 occurred in Patient 22 even with a supratentorial tumor; the cumulative cochlea RT dose was estimated as being the same as the CSI dose (18 Gy). Age at the start of RT (≥5 years vs. <5 years) did not influence Brock grades ≤1 or ≥2. Patients 5 and 14 exhibited hearing loss of Brock grades 2 and 4, respectively, just after surgery, because their tumors were located close to the auditory nerve.

### Cognitive functions

We used three different types of IQ test, which included the Wechsler Intelligence Scale for Children-III, the Tanaka-Binet Intelligence Scale, and the Kyoto Scale of Psychological Development 2001. Appropriate tests were used dependent on patient age.

**Figure 5:**
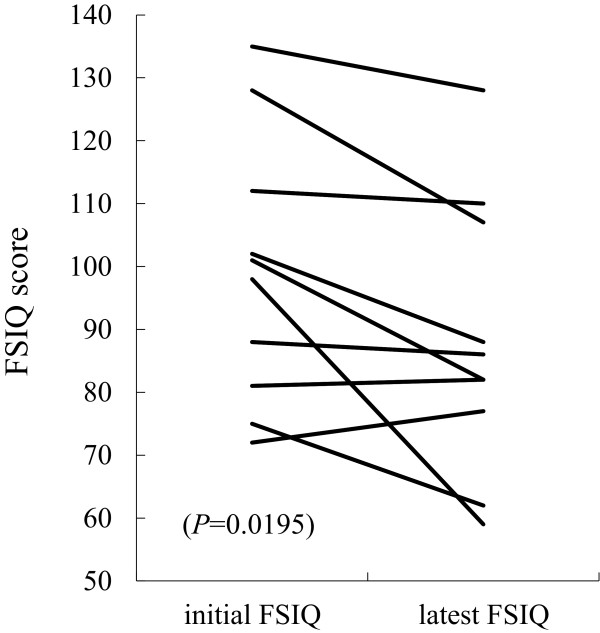
**Differences in the full-scale intelligence quotient (FSIQ) at diagnosis and at the last assessment.**

IQ tests were performed after RT on 17 of the 24 patients. Ten of the 17 patients had multiple/repeated IQ tests at least twice during follow-up. The data for each patient is presented in Tables 1 and 2. The median time between the initial and latest IQ tests was 31 (range, 5–75) months. The differences in these scores were significant and in the range of 5 to -39 (median, -10; *P* = 0.0195) (Figure 5). The median latest full-scale IQ (FSIQ) score was 86 (range, 59–128). In 13 of the 17 patients, these scores were <100. Patients 3 and 22 had a FSIQ <70 at the last assessment, which represented severe mental retardation. Neither the age at the start of RT (≥5 years vs. <5 years) nor the primary tumor site (supra- or infra-tentorial) nor the CSI dose (>18 Gy vs. ≤18 Gy) influenced the FSIQ score.

All of the 18 patients who were disease free until the last assessment attended normal school. However, six of the 18 patients had learning disabilities.

### Miscellaneous findings

Patient 2 developed a bilateral cataract at 2 years after RT, but surgery has not yet been required. Patient 14 developed obesity and hyperlipidemia. She was suspected as having hypothalamic obesity. Patient 20 was suspected of precocious puberty. Patient 23 developed Moyamoya disease at 2 years after RT. He had encephaloduroarteriosynangiosis; however, he developed right hemiparesis caused by cerebral infarction after surgery. No secondary malignant disease has been reported in patients investigated in this study.

## Discussion

In the present study, we used a reduced-dose (18 or 23.4 Gy) CSI protocol for the treatment of all embryonal CNS tumor patients with M0 disease. Generally, this protocol has been applied for limited to average-risk MB patients. For the treatment of high-risk M0 patients, we administered aggressive chemotherapy to compensate for the reduced-dose CSI treatment. As a result, the PFS and OS rates were not significantly different between the average- and high-risk groups. For patients with high-risk MB, studies during the 1990s typically showed a 5-year PFS of 40–50% following CSI at approximately 36 Gy and chemotherapy [6, 19]. In the present study, the treatment with reduced-dose CSI for the high-risk MB patients resulted in 5-year PFS and OS rates of 85.7 and 83.3%, respectively; these rates did not differ significantly from those for average-risk MB patients. In addition, several studies reported that sPNET has a poorer prognosis than high-risk MB [20, 21]. In the case of sPNET, all four patients had M0 disease and three of them remained disease free. These results are promising, although the patient cohort was small and requires further follow-up.

Some data have previously been reported regarding the efficacy of CSI at a dose of 18 Gy instead of 23.4 Gy for younger patients [22, 23]. In our study, all M0 patients aged <5 years received reduced-dose CSI (18 Gy) independent of tumor type. There were no differences in the 5-year OS and PFS rates between patient groups after CSI at doses of 18 and 23.4 Gy. In particular, four sPNET and one AT/RT patient with M0 disease received CSI at 18 Gy, and four of these patients remained disease free. It is possible that CSI at 18 Gy may be feasible and yield favorable clinical outcomes for young age groups. In addition, CSI at this dose may reduce adverse effects in young patients, as discussed later. Moreover, it should be established in further clinical trials if a CSI dose of 18 Gy would also be sufficient for older patients. The current Children’s Oncology Group study is testing the efficacy of CSI (18 Gy) in the treatment of average-risk MB patients aged 3–8 years.

In AT/RT, leptomeningeal dissemination is often observed and the outcome is worse than that for other embryonal tumors. According to recent estimates, the median OS is in the range of 17–48 months [24–26]. In our study, two of three patients with AT/RT had M3 disease and died despite aggressive therapies including HDC with ASCT and RT. Our protocol was not effective in the treatment of AT/RT with M3 disease, and further consideration of treatment strategy is required.

The dilemma regarding the treatment of AT/RT is as follows. Many patients are younger than 3 years at diagnosis [27], which is associated with poorer prognosis [12]. However, it is difficult to decide whether RT should be administered to patients in this age range who are at risk of serious late adverse effects. Recent studies have reported that RT might be more effective than chemotherapy as an adjuvant treatment, even for patients younger than 3 years [26, 28]. For example, Chi et al. [29] have shown that the combination of intrathecal chemotherapy with RT delivered exclusively to the tumor bed may be a potentially effective strategy for disease control in these younger patients. In our study, younger patients with M3 disease were the most difficult to cure. Whether or not this new strategy is also effective for this patient group has not yet been clarified; novel clinical trials might be worthwhile.

There have been many studies regarding height impairment in patients with embryonal CNS tumors treated with CSI [30–32]. Height impairment in these patients is attributed to a combination of growth hormone deficiency and radiation osteitis of the vertebral column. Adan et al. [33] reported that the final height SDSs in patients who received CSI at doses of 32–36 Gy ranged from -2.4 to -1.6; in addition, height impairment in young patients was found to be severe. Kiltie et al. [34] reported that final height SDSs in patients with MB aged < 3 years who received CSI at 27–30 Gy was -4.2. In our study, the median height SDS at the last assessment was -1.6; in particular, that of younger patients who were < 5 years old was -1.9. This value was less serious than those reported in the previous studies mentioned above. The possible reasons of our favorable results were that our younger M0 patients received lower CSI doses (18 Gy). Brownstein et al. [32] reported that final height and loss of height SDSs were negatively associated with CSI dose (<20 vs. ≥20 Gy; *P* < 0.001). Xu et al. [35] reported that patients who received CSI at 18 Gy were significantly taller than those who received CSI at 23–39 Gy. Thus, CSI at 18 Gy in young patients may have beneficial effects regarding height. It will be necessary to continue long-term follow-up because most of our patients had not reached their final height at the last assessment.

Hypothyroidism is a well-documented common late effect associated with CSI. In addition, hypothyroidism is more common in children who received chemotherapy and CSI [36, 37]. Moreover, survivors of pediatric hematopoietic stem cell transplantation are at increased risk of thyroid dysfunction, after total body irradiation at a dose of >7.5 Gy [38]. In our study, hypothyroidism occurred even in two patients who had received CSI at 18 Gy (Patients 1 and 19); according to retrospective assessment of the CT planning data, the mean dose to the thyroid gland was around 12 Gy. It is important to take care to reduce the dose to the thyroid gland by means of patient positioning and treatment planning; for example, it could be effective to set the cranial-spinal junction as low as possible.

It has been reported that loss of high-frequency sensorineural hearing is induced in children by both cisplatin and radiation [17, 39, 40]. In RT-induced ototoxicity, the severity of hearing loss is related to the dose to the cochlea. Hua et al. [40] and Merchant et al. [18] suggested that the mean cochlear dose should be less than 35 and 32 Gy, respectively. In our study, 50% of patients suffered hearing loss of Brock grade ≥2. Of note, we found that a patient with supratentorial tumor exhibited hearing loss, while the cumulative cochlea RT dose was as low as the CSI dose (18 Gy) (Patient 22). Taken together, the threshold dose has not been established in pediatric patients. In any case, the dose to the cochlea should be reduced as much as possible. New RT modalities (i.e., intensity-modulated RT and proton therapy) may avoid the adverse radiation effects on the thyroid gland and on the cochlea.

Cognitive dysfunction is common in patients treated with cranial RT. Several previous studies have shown that young age is the most prominent risk factor, and that radiation dose can also be used to predict a decline in the IQ score [41, 42]. In our study, while the latest FSIQ was lower than 100 in many patients (76.5%), neither the age at the start of RT nor the primary tumor site influenced the FSIQ score. These discrepancies may have occurred as a result of the fact that the younger M0 patients (<5 years at the start of RT) in our study received lower CSI doses (18 Gy) than those given in previous studies. In addition, all survivors have attended normal school. Our data suggested that reduced-dose CSI for young children may have beneficial effects regarding cognitive functions.

## Conclusions

In conclusion, our protocol for patients with embryonal CNS tumors was feasible and yielded favorable clinical outcomes. The PFS and OS rates were not significantly different between the average- and high-risk groups, not only in the case of MB but also other embryonal CNS tumors. While M0 patients aged <5 years received reduced-dose CSI at 18 Gy, their treatment outcomes, growth heights, and cognitive functions were the same as those for other patients. It is possible that CSI at 18 Gy may be feasible and yield favorable clinical outcomes for young age groups. Although there were no life-threatening or disabling late toxicities, several adverse effects that affected quality of life were observed. Close follow-up and appropriate managements will be essential for all patients with embryonal CNS tumors.

## Abbreviations

CNS: Central nervous system
MB: Medulloblastoma
sPNET: Supratentorial primitive neuroectodermal tumor
CSI: Craniospinal irradiation
PFS: Progression-free survival
OS: Overall survival
SDS: Standard deviation score
FSIQ: Full-scale intelligence quotient
AT/RT: Atypical teratoid/rhabdoid tumor
RT: Radiation therapy
CCSG: Children's Cancer Study Group
HDC: High-dose chemotherapy
ASCT: Autologous stem-cell transplantation
MRI: Magnetic resonance imaging
GTR: Gross total resection
VVCC: Vincristine, etoposide, cisplatin, and cyclophosphamide
MV: Megavolt
IQ: Intelligence quotient
T4: Thyroxine
TSH: Thyroid-stimulating hormone.
